# Cerebrovascular Reactivity Assays Collateral Function in Carotid Stenosis

**DOI:** 10.3389/fphys.2020.01031

**Published:** 2020-09-01

**Authors:** Olivia Sobczyk, Kevin Sam, Daniel M. Mandell, Adrian P. Crawley, Lakshmikumar Venkatraghavan, Larissa McKetton, Julien Poublanc, James Duffin, Joseph A. Fisher, David J. Mikulis

**Affiliations:** ^1^ Institute of Medical Science, University of Toronto, Toronto, ON, Canada; ^2^ Joint Department of Medical Imaging and the Functional Neuroimaging Laboratory, University Health Network, Toronto, ON, Canada; ^3^ Department of Anaesthesia and Pain Management, University Health Network, Toronto, ON, Canada; ^4^ Department of Physiology, University of Toronto, Toronto, ON, Canada

**Keywords:** cerebrovascular reactivity, collaterals, cerebral blood flow, cerebrovascular disease, stroke, MRI

## Abstract

In patients with carotid artery stenosis (CAS), the risk of stroke, its severity, and response to revascularization are strongly related to the availability of collateral blood flow. Unfortunately, there is poor agreement between observers in assessing collateral flow using flow-based imaging. We used changes in blood-oxygen-level-dependent (BOLD) MRI as a surrogate of changes in regional cerebral blood flow in response to a hypercapnic stimulus [i.e., cerebrovascular reactivity (CVR)] as indicating flow reserve ipsilateral to CAS. We hypothesized that some patients with hemodynamically significant CAS develop functional collateral flow as indicated by normalization of ipsilateral CVR. We identified 55 patients in our CVR database with various degrees of CAS assessed by angiography and classed them as <50% stenosis, 50–69% stenosis, 70–90% stenosis, >90% stenosis, and full occlusion. CVR was measured as the change in BOLD signal in response to changes in end-tidal partial pressure of CO_2_ (Δ BOLD/Δ PETCO_2_) and normalized voxel-wise relative to the mean and standard deviation of the CVR in the corresponding voxels of an atlas of 46 healthy controls (CVR z scores). CVR and z scores were then averaged over gray matter (GM) and white matter (WM) on each side of the middle cerebral artery (MCA) territory. As hypothesized, CVR varied for each severity of CAS. Ipsilateral MCA territory CVR was less than normal in each class, including that with <50% stenosis (Student *t*-test, two-tailed; *p* = 0.0014 for GM and *p* = 0.030 for WM), with a trend of decreasing average CVR with increasing stenosis. Remarkably, the considerable individual variability in MCA CVR included some patients with normal CVR in each class – including that with complete occlusion. We conclude that, in general, CAS depresses downstream vascular reserve, but the extent of collateralization is highly variable and not predictable from the degree of stenosis, including both <50% stenosis and complete occlusion. CVR may be the more reliable marker for recruitable collateral blood flow than degree of CAS.

## Introduction

Stroke is the leading cause of disability and the third leading cause of death in North America, with approximately 85% of strokes ischemic in nature (Writing Group Members et al., 2016). Nevertheless, even among symptomatic patients with severe carotid artery stenosis (CAS) or occlusion, stroke incidence is only 12–15% in the first year following diagnosis, indicating the possibility that high and low risk subgroups exist among these patients (Blaser et al., 2002; Amarenco et al., 2018). Patients with CAS may undergo risk stratification based on the severity and frequency of symptoms, morphology of the lesion (Cogswell et al., 2017; Mandell et al., 2017), and Doppler or angiographic assessment of the degree of stenosis (Derdeyn et al., 2002; Brott et al., 2010). Other than morphology of the lesion, most of these parameters are poorly related to the risk of stroke (Gupta et al., 2012; Reinhard et al., 2014). It has become clear over the last decade that the co-existence of *recruitable* collateral blood flow (Bang et al., 2008; Sheth et al., 2016; Tan et al., 2016) is one of the strongest predictors of stroke risk, in terms of its severity, prognosis (Lima et al., 2010; Menon et al., 2011), and response to therapy. Current tests of cerebral perfusion (CT and MR perfusion imaging) assess resting blood flow but provide no information about the availability of distal recruitable perfusion (Powers, 1991), i.e., collateral blood flow function, to compensate for an upstream flow obstruction such as from a clot or embolism (Fanou et al., 2015; Leng et al., 2016).

As an alternative to having a reliable and quantifiable image of collateral flow (Ben Hassen et al., 2019), a functional assay, such as the flow response to an increased demand, is required. An increased arterial partial pressure of carbon dioxide (“hypercapnia”) can provide a strong global vasostimulation. Changes in MRI blood-oxygen-level-dependent (BOLD) signal within an interrogated voxel arise from changes in capillary flow and venules and are, therefore, good markers for local tissue blood flow responses (Ogawa et al., 1993; Hoge et al., 1999). The ratio of the percent change in BOLD signal, in response to a given hypercapnic stimulus, is called cerebrovascular reactivity (CVR).

All cerebral vascular beds dilate in response to hypercapnia, causing them to compete for flow from the major extracranial arteries (Sobczyk et al., 2014). In health, the changes in vascular resistance in response to hypercapnia are balanced, and the hyperemia is uniformly distributed throughout the brain (Bhogal et al., 2016). However, in the case of an uncompensated hemodynamically significant unilateral CAS, hypercapnia increases flow on the healthy side, reducing the overall perfusion pressure, and thus the flow past the stenosis. This discrepancy in flows is reflected in the BOLD signal in the respective hemispheres (Sobczyk et al., 2014). On the other hand, if recruitable collateral flow is present on the side of CAS, there is an increase in flow and, therefore, in the BOLD signal. This assumption of the significance of increase in BOLD arises from the observation that the strength of ipsilateral CVR has a strong inverse correlation with the risk of stroke regardless of the degree of stenosis (Gupta et al., 2012; Reinhard et al., 2014).

Nevertheless, “degree of stenosis” and “hemodynamic compromise” continue to be dominant indications for therapeutic interventions and have been adopted as the chief inclusion criterion in trials such as CREST2 (Howard et al., 2017). To obtain insight into which criterion our own data support, we reviewed our existing clinical CVR database to find the relationship between the radiologically-measured severity of CAS and the presence of recruitable blood flow as indicated by CVR. We hypothesized that the hemodynamic severity of the CAS will be weakly associated with parenchymal CVR, implying that in some patients, the hemodynamic restrictions at the site of the lesion can be offset by functioning collaterals.

## Materials and Methods

### Subjects and Ethical Approval

All studies conformed to the standards set by the latest revision of the Declaration of Helsinki and were approved by the Research Ethics Board of the University Health Network and Health Canada. Subjects were participants in prospective studies in which CVR was measured in healthy subjects and those with cerebral vascular disease referred for investigation of neurological symptoms. All subjects provided written and informed consent for the original studies.

This study followed the principle of standardized uniform stimulus and data post-processing (Sobczyk et al., 2015, 2016) to optimize the reproducibility of data between patients (Smeeing et al., 2016). The data are presented normalized to a healthy cohort, thus enabling comparisons to data generated at other centers. Normative data were generated from studies in 46 healthy individuals with no history of neurological or cardiovascular disease and who were non-smokers and taking no medication, as previously described (Sobczyk et al., 2015). For study subjects, we searched our CVR research database for all adult patients of either sex with known CAS disease to any exent but who had come to medical attention as a result of transient symptoms and had not suffered a cerebral infarction at the time of initial study. This search yeilded 55 individuals (demographics listed in Supplementary Table 1). The grade of stenosis was assessed by two staff neuroradiologists reviewing all available angiograms [magnetic resonance angiography (MRA), computed tomography angiogram (CTA), and catheter angiograms] and categorized as: Class 1, none; Class 2, <50%; Class 3, 50–69%; Class 4, 70–90%; Class 5, >90%; and Class 6, 100%.

### Experimental Protocol

#### Hypercapnic Stimulus

Subjects underwent a standardized iso-oxic hypercapnic stimulus using an automated gas blender (RespirAct™, Thornhill Research Inc., Toronto, Canada) running a prospective gas targeting algorithm (Slessarev et al., 2007). The stimulus consisted of two step increases of 10 mmHg from a baseline end-tidal partial pressure of carbon dioxide (PetCO_2_) of 40 mmHg, lasting 90 and 120 s (Fierstra et al., 2013; Fisher et al., 2018).

#### MRI Acquisition and CVR Map Generation

Magnetic resonance imaging was performed with a 3.0-Tesla scanner (Signa HDx; GE Healthcare, Milwaukee, Wisconsin) with an 8-channel phased-array head coil and consisted of BOLD acquisitions using a gradient echo pulse sequence with echoplanar readout (TR/TE = 2000/30 ms, field of view 24 cm × 24 cm, matrix size 64 × 64, number of temporal frames 254, 39 slices, slice thickness 5 mm, no interslice gap, and flip angle 85°). The acquired MRI and PetCO_2_ data were analyzed using Analysis of Functional NeuroImages software (AFNI^1^; Cox, 1996). PetCO_2_ data were time-shifted to the point of maximum correlation with the voxel showing the greatest positive BOLD signal change and re-sampled at the repetition time. A linear, least-squares fit of the BOLD signal data series to the PetCO_2_ data series was then performed on a voxel-by-voxel basis. BOLD images were then volume registered and slice-time corrected and co-registered to an axial 3-D T1-weighted Inversion-Recovery Prepared Fast Spoiled Gradient-Echo (IR-FSPGR) volume (TI/TR/TE = 450/8/3 ms, matrix size 256 × 256, field of view 22 cm × 22 cm, slice thickness 1 mm, and flip angle 15°) that was acquired in the same imaging session. CVR maps were generated by color-coding the slope of the linear relation between the change in %BOLD signal and the corresponding change of PetCO_2_ to a spectrum of colors reflecting the direction and magnitude of the CVR and overlaid on the corresponding structural scans. All voxels with correlation coefficients between −0.125 and +0.125 were thresholded from the maps.

### Analysis of CVR Data

**Figure 1:**
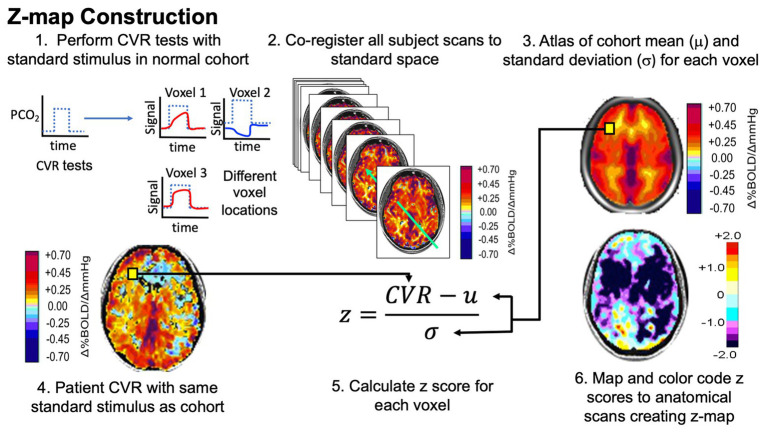
Pathway to normalizing cerebrovascular reactivity (CVR). Narrative description in text. Figure modified with permission from Fisher et al. (2018).

This methodology is described in detail in Sobczyk et al. (2015) and summarized below and in Figure 1.

#### Atlas Construction and z Scores

Analytical processing software (SPM8; Wellcome Department of Imaging Neuroscience, University College, London, UK^2^) was used to co-register each of the individual brain volumes from the normative data (Sobczyk et al., 2015) into Montreal Neurologic Institute (MNI) standard space, and the mean CVR and associated standard deviation (SD) were calculated for each voxel across all 46 healthy subjects (AFNI software; Cox, 1996) to generate a normal atlas. Individual z scores were then calculated on a voxel-wise basis by co-registering their CVR maps to the same space as the atlas and scoring each subject’s CVR value by assigning a z score according to the mean and SD of the corresponding voxel of the atlas. The healthy subjects’ individual z scores were calculated by a “leave one out” or “jackknife” procedure, which required taking each healthy subject from the reference atlas and z scoring that subject to the remaining atlas (Minoshima et al., 1995).

#### Segmentation

The co-registered T1-weighted anatomical images for all subjects were segmented into gray and white matters (GM and WM) using the aforementioned SMP8 software and further segmented into right and left middle cerebral artery (MCA) territories using masks created manually by a neuroradiologist (DMM). For each subject, hemispheric GM and WM mean z scores were calculated for the MCA territory for each side.

#### Statistical Analysis

The internal carotid artery (ICA) stenosis/occlusions measures for each hemisphere were divided into groups based on the degree of stenosis classification mentioned earlier. For each group, the proportion of hemispheric mean MCA z scores compared to the entire combined group mean MCA z scores was calculated and their distribution displayed in frequency distribution histograms (FDH). Statistical analysis was performed with SigmaPlot 11.0 software package (SigmaPlot version 12, Systat Software, Inc., San Jose California^3^). Student *t*-tests (two-sided, *α* = 0.05) were performed between categories to determine if significant differences existed between the groups. To examine the effect of hemispheres with high grade stenosis (>50% stenosis) on hemispheres with <50% stenosis, the mean hemispheric z scores were re-categorized in the follow manner: Group A is hemispheres with both <50% stenosis, Group B is the hemispheres with ≥50% stenosis with a contralateral side having <50% stenosis, and Group C is hemispheres with both ≥50% stenosis. The distributions of these categories were plotted in FDH, and a one-way analysis of variance (ANOVA) with the Holm-Sidak method of multiple comparisons correction was performed to determine if any significant differences existed between the three groups (*α* = 0.05). Using percent stenosis as a threshold, receiver-operator (ROC) curves were generated for hemispheric z scores in GM and WM.

## Results

**Figure 2:**
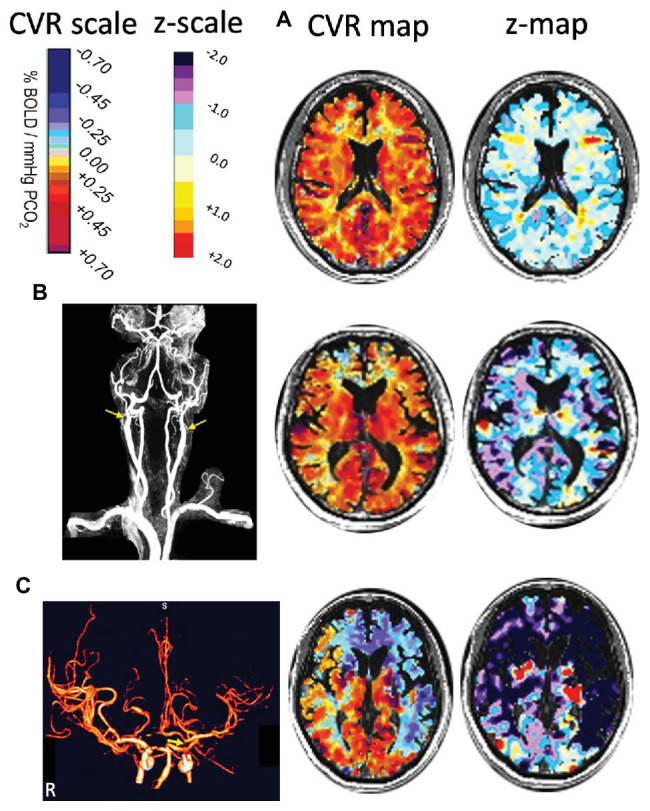
Three cases illustrating how z-maps display abnormality not readily distinguished from CVR maps. **(A)** A healthy 65-year old control subject. The CVR and z-maps of a representative axial slice show that the distribution of z scores are around 0 within a range of ±1 SD. **(B)** A 76-year-old man with bilateral proximal internal carotid artery (ICA) occlusions (as shown by arrows in MRA) and normal neurological examination. The CVR and z-maps of a representative axial slice show that the spatial distribution of CVR values was restricted to the lower normal range compared to the CVR z-map in A. **(C)** A 59-year-old man with transient ischemic attacks but no MRI evidence of ischemic injury. CT angiography shows distal left ICA near occlusion (arrow) and less than 50% proximal right ICA stenosis (not shown). The CVR and z-maps of a representative axial slice show steal (blue CVR) in the RACA, LACA, and LMCA territories. Note the absent right A1 segment of the RACA accounting for steal caused by the distal LICA stenosis, with the z-map measuring the severity and extent of vascular abnormality. CT, computed tomography; CVR, cerebrovascular reactivity; LACA, left anterior cerebral artery; LICA, left internal carotid artery; LMCA, left middle cerebral artery; MRA, magnetic resonance angiography; RACA, right anterior cerebral artery.

The anthropomorphic characteristics of the control group have been reported in Sobczyk et al. (2015). A representative control subject’s (65-year-old male) MRA, CVR, and corresponding z-map are shown in Figure 2A.

The patient group consisted of 41 males and 14 females with an average age of 61 ± 17 years (mean ± SD). Patient details, diagnoses and mean CVR and z score results are listed in Supplementary Table 1. In summary, there were 40 hemispheres with <50% stenosis, 11 with 50–69% stenosis, 12 with ≥70% stenosis, 7 with >90% ICA stenosis, and 40 hemispheres with complete ICA occlusion.

**Figure 3:**
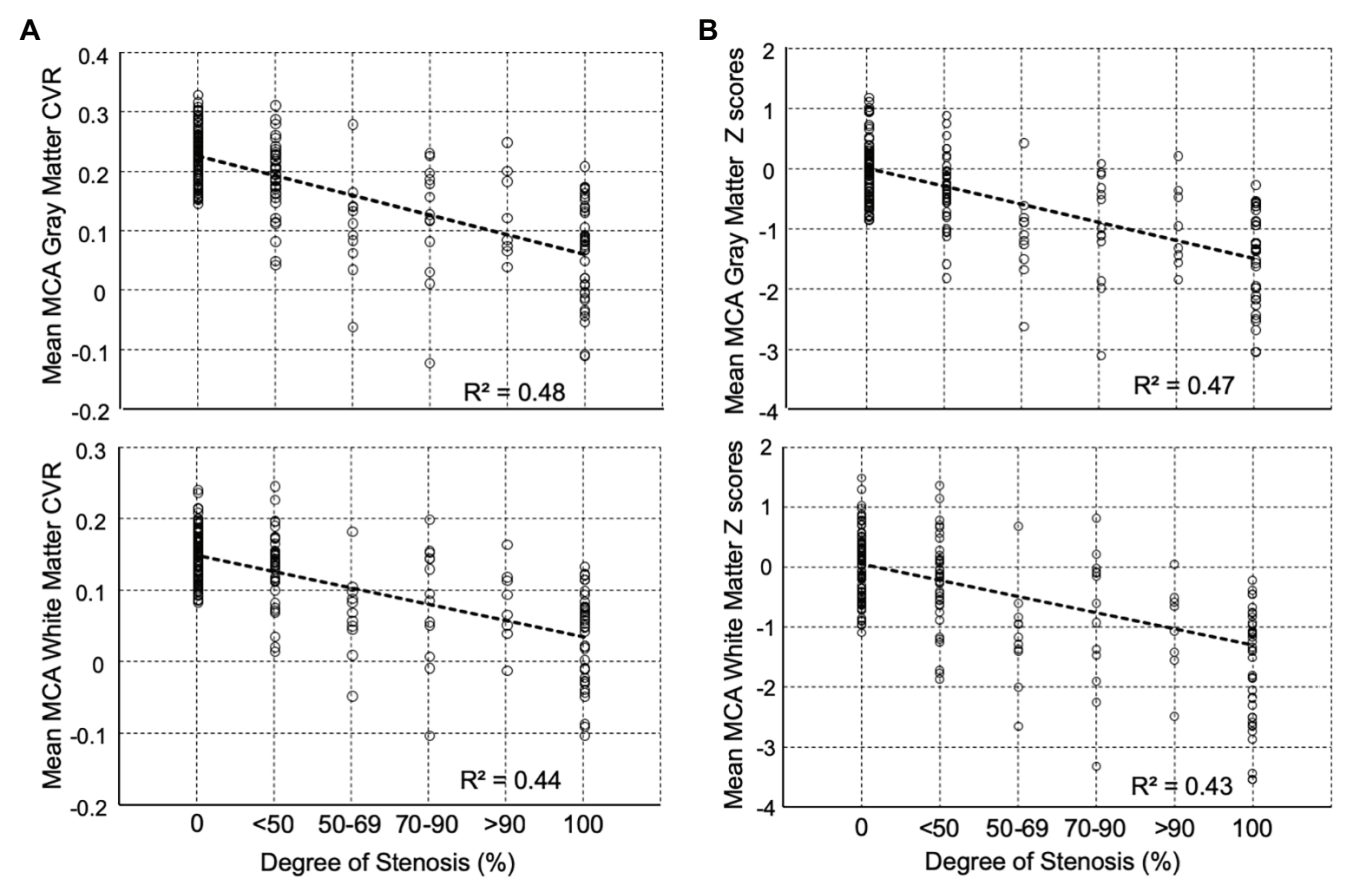
Distribution of mean middle cerebral artery (MCA) percent stenosis category for **(A)** CVR (Δ% BOLD/Δ mmHg PetCO_2_) and **(B)** z scores for both gray and white matters. Beyond 50% stenosis, the distribution of CVR no longer has the distribution of the control cohort. All pairwise multiple comparison rank test (Dunn’s Method) showed for gray matter that all groups differed from the control group (*p* < 0.05) except <50% stenosis group, and for white matter all groups were significantly different from the control group (*p* < 0.05) except for the <50% stenosis and 70–90% stenosis groups. Note: Abscissas in B show average z scores for all voxels in the MCA region of interest (ROI). In an individual subject, CVR values in a voxel may contain errors due to partial voluming, location misregistration, subject movement, position, and others; however, when all the voxels are averaged over a large ROI, they more truly represent the underlying physiology of the ROI. The ROI z score of individual healthy subjects was calculated by performing a “jackknife” procedure, which involved taking each healthy subject from the reference atlas, calculating a mean CVR and comparing it back to the corresponding atlas average CVR. The range of z scores in the healthy individuals (comparable to the standard error of the mean) in the atlas was ±1. CVR, cerebrovascular reactivity; BOLD, blood-oxygen-level-dependent; PetCO_2_, end-tidal partial pressure of carbon dioxide.

Differences in mean MCA, CVR, and z scores between the percent stenosis categories are displayed in Figure 3 for both GM and WM. The graphs present a relationship between percent stenosis and mean CVR and z scores. The z scores are a standardized score accounting for measurement and normal physiological variability (Sobczyk et al., 2015). The trend shows that the greater the stenosis the lower the mean CVR and z scores. However, at each percent stenosis, the range of CVR overlapped the normal range but only for its lower half, with mean z scores between 0 and −1; there were no z scores between 0 and +1. This observation is consistent with the principle of incomplete collateral flow compensation. Detailed imaging from one of these patients with severe CAS and near normal CVR is shown in Figure 2B.

**Figure 4:**
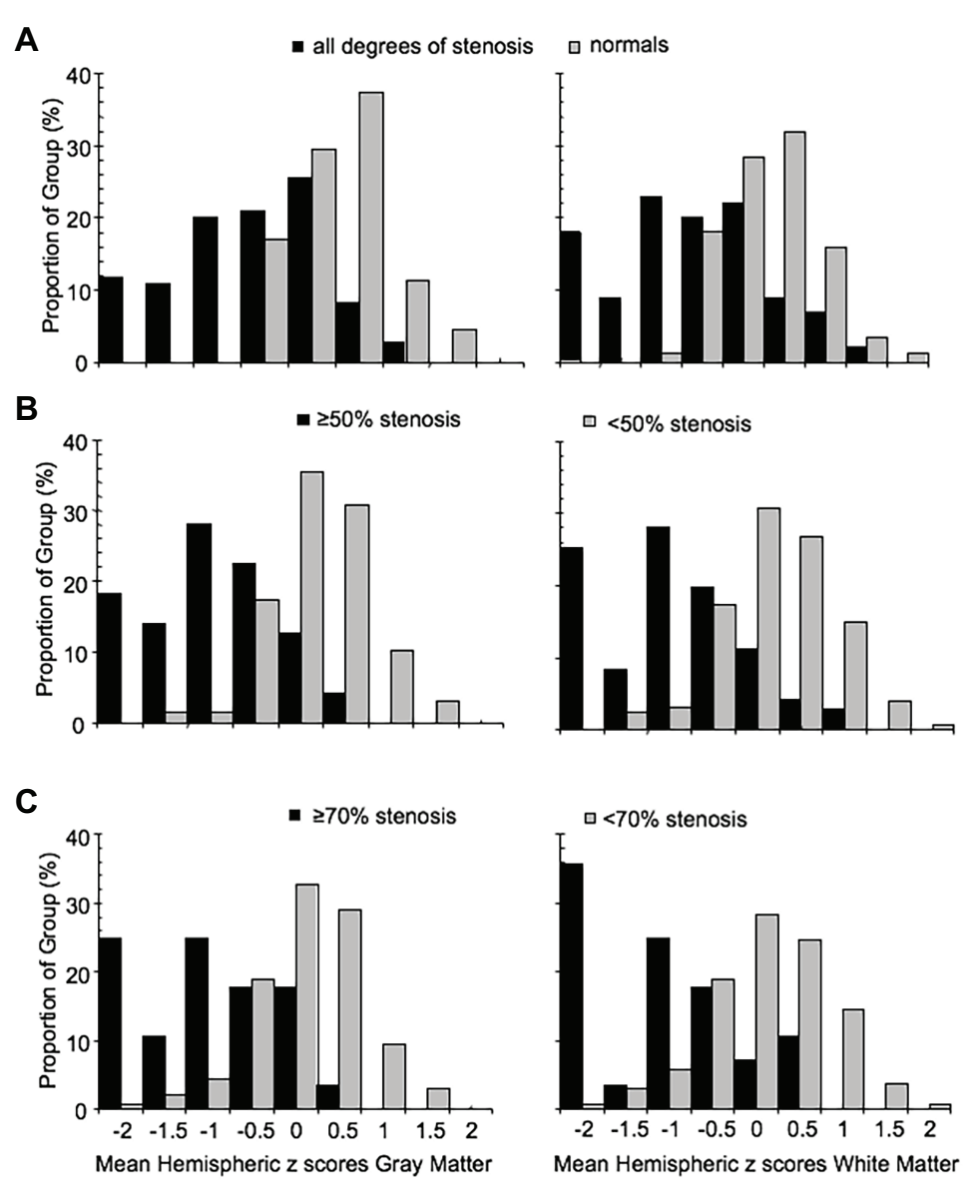
Frequency distribution histograms of the mean MCA hemispheric z scores of gray and white matter for **(A)** normal group and all ICA stenosis groups regardless of degree of stenosis, **(B)** hemispheres with ICA <50% stenosis (including normal group) and hemispheres with ≥50% stenosis, and **(C)** hemispheres with ICA <70% stenosis and hemispheres with ICA ≥70% stenosis. ICA, internal carotid artery; MCA, middle cerebral artery.

The distribution of z scores for GM and WM, based on the degree of stenosis, is shown in Figure 4. There was a statistically significant difference in CVR between the control group and patients, regardless of degree of stenosis, including those with <50% stenosis for both GM and WM (*p* = 0.0014 for GM and *p* = 0.030 for WM). Significant differences were also present between the <50% stenosis group and 50–69% stenosis group in both GM and WM (*p* < 0.001 for GM and *p* = 0.001 WM). There were no significant differences (*p* = 0.409 for GM and *p* = 0.38 for WM) between the remaining hemispheric groups. A number of hemispheres with <50% ICA stenosis had reduced CVR outside the normal distribution; an example of a patient in this category with bilateral carotid stenosis is shown in Figure 2C.

**Figure 5:**
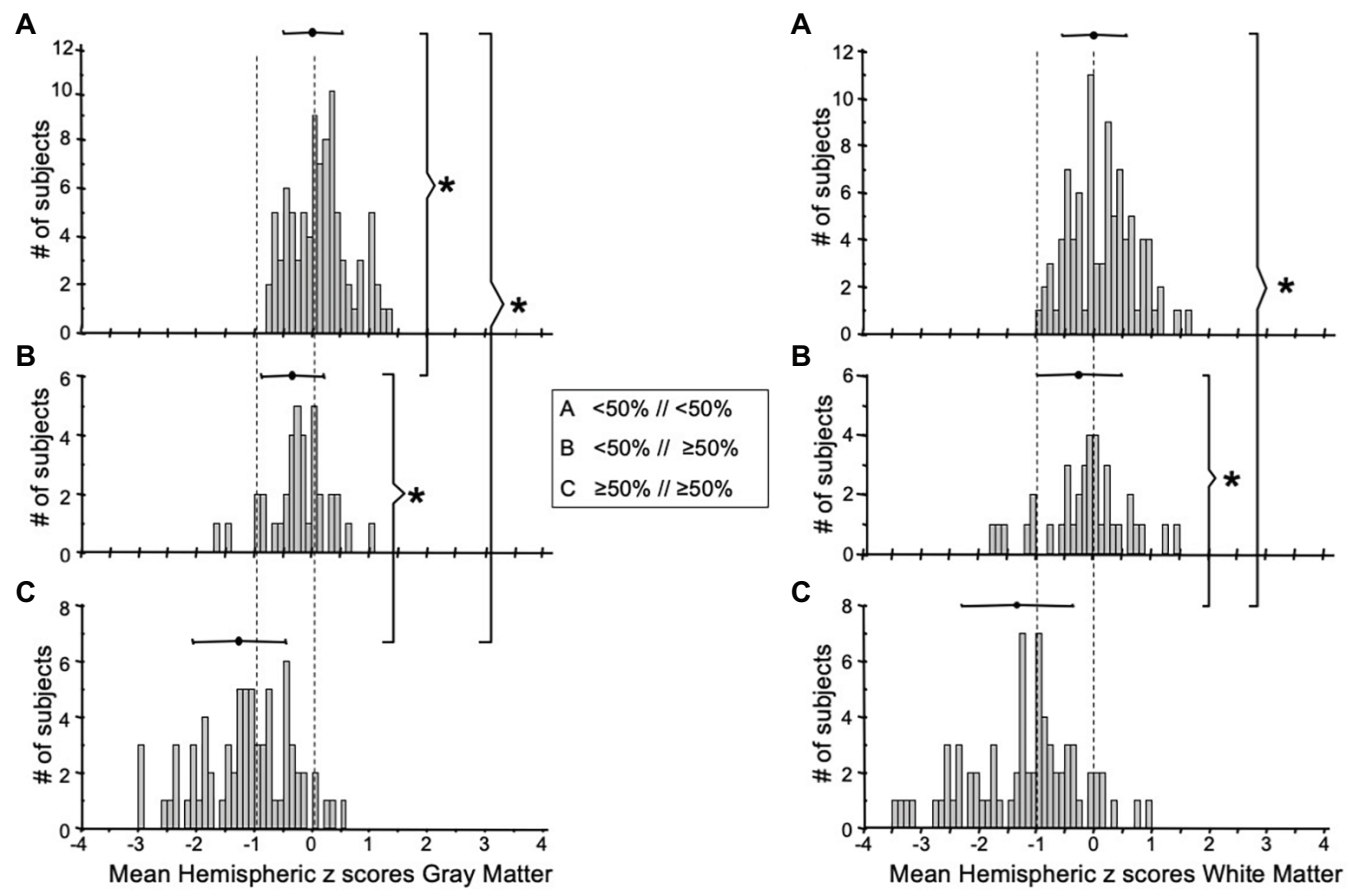
FDH of the mean MCA hemispheric z scores distribution of gray (left side graphs) and white matter (right side graphs) with **(A)** both hemispheres <50% stenosis, **(B)** one hemisphere ≥50% stenosis with the other hemisphere <50% stenosis, and **(C)** both hemispheres with ≥50% stenosis. ^*^Indicates significantly different distributions. FDH, frequency distribution histograms; MCA, middle cerebral artery.

It has been found that the CVR of the contralateral side is affected to a limited degree, by the ipsilateral side with high-grade stenosis (Sam et al., 2014). We have also observed subjects exhibiting this pattern from our cohort (Figure 2C). To examine the effect of high grade stenosis on the contralateral side of our cohorts, the mean hemispheric z scores were re-categorized in the follow manner: Group A is both hemispheres with <50% stenosis, Group B is the hemispheres with ≥50% stenosis with a contralateral side <50% stenosis, and Group C is both hemispheres with ≥50% stenosis. The distributions of these categories are found in the FDH in Figure 5. One-way ANOVA with Holm-Sidak method of multiple comparisons correction found significant differences between all three groups for both GM and WM (*p* < 0.001, *α* = 0.05) excluding WM Group A vs. Group B, which showed no significant differences after multiple comparison correction.

**Figure 6:**
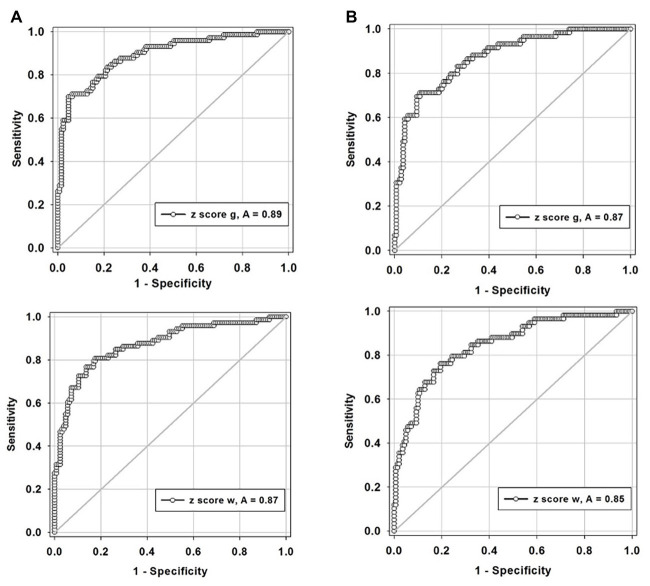
Receiver-operator curves displaying the accuracy of degree of stenosis as a predictor for hemodynamic impairment for, **(A)** defining any hemisphere with ICA ≥50% stenosis in both gray and white matters and, **(B)** defining any hemisphere with ICA ≥70% stenosis in both gray (g, gray matter; top graphs) and white matter (w, white matter; lower graphs). ICA, internal carotid artery.

Two criteria were examined for defining “abnormal”: (1) any hemisphere with ICA ≥50% stenosis and (2) any hemisphere with ICA ≥70% stenosis. The ROC curves (Figure 6) demonstrate that this standardized CVR test is able to identify those with ≥50% stenosis who have impaired CVR [area-under-the curve (AUC)] for GM was 0.89 (95% *CI* = 0.85–0.94) and 0.87 (95% *CI* = 0.82–0.93) for WM. This indicates that the using two criteria to define hemispheres with ICA ≥50 and ≥70% stenosis as abnormal made little difference. False negatives (CVR in the normal range) were found in the hemispheres of patients with normal CVR due to sufficient collateral blood flow, regardless of the presence of a high-grade stenosis; presented in Figure 2B. However, Figure 3 shows that those hemispheres within the normal range for CVR do not have the same distribution as normal hemispheres in healthy subjects. They lack the CVR in healthy subjects that have z scores >0.

## Discussion

### Main Findings

In this study, we demonstrate a progressive decrease in CVR associated with increasing degree of CAS. However, the finding with the most far-reaching implication is that some patients with the more severe stenoses – including those with complete occlusion – had near normal ipsilateral CVR. The increase in the BOLD signal results from increased cerebral blood flow (CBF) and resulting reduction in deoxyhemoglobin concentration (Hoge et al., 1999). This surge of flow must be routed around the upstream stenosis, arriving through collateral pathways *via* the circle of Willis, or further downstream through interconnections between pial vessels, penetrating arterioles and capillaries (Fisher et al., 2018). The corollary implication is that patients with ipsilateral reductions in BOLD response lacked a similar extent of collateralization, and therefore, perfusion is unable to circumvent the stenotic part of the feeding vessel.

### Steady State Vs. Responsive Cerebral Blood Flow

A perspective of these finding can be obtained by comparing them to those of Powers (1991). In that study, as in ours, patients had varying degrees of stenosis but no clinical or radiological signs of stroke. They were studied with positron emission tomography (PET) to determine resting CBF and oxygen extraction fraction (OEF). In Powers Stage 1 ischemia, near normal CBF and OEF are sustained by vasodilation, collateral blood flow, or both. PET measures the net brain blood flow but cannot determine the relative contributions of the two mechanisms, much less the *reserve vasodilatory capacity*. The latter requires a provocative vasoactive stimulus and a measure of a change in blood flow, both features of CVR. Flow is not measured directly but the change in the BOLD signal closely follows the magnitude of the change in flow (Hoge et al., 1999), making CVR a semi-quantitative measure. Finally, we note that whereas CVR provides a unique assessment of each patient, the model of Powers (1991) is based on group trends which do not address the risks in particular individuals, a requirement for clinical practice.

### Standard Measures of Vascular Perfusion Integrity

Vascular perfusion integrity can be tested clinically. Digital subtraction catheter angiography has been used to generate angiographic images but the method is limited by lacking a standardized scoring method (McVerry et al., 2012). The American Society of Interventional and Therapeutic Neuroradiology/Society of Interventional Radiology (ASITN/SIR) collateral score was developed to harmonize collateral circulation grading for CTA or perfusion MRI-based methods (McVerry et al., 2012; Ben Hassen et al., 2019) and is currently the recommended and the most commonly used scale in endovascular acute stroke trials for measuring collateral flow and decision-making on mechanical thrombectomy (Powers et al., 2019). Ben Hassen et al. (2019) tested the best case for inter‐ and intra-observer agreement of this scale in a randomized control trial, picking trained readers selected from interventional neuroradiologists who enrolled patients in the THRACE trial (Bracard et al., 2016). They provided the readers with a pre-trial training set and tested them on 30 cases selected for high image quality, showing opacification of ipsilateral posterior cerebral artery that enabled temporal territory evaluation. The overall agreement on a four-point scale was rated as “poor,” improving only slightly when the scale was dichotomized or readers grouped by years of experience. The intra-observer agreement varied greatly between readers, without a consistent pattern such as relation to experience.

In contrast, in two metanalyses examining measures correlating with the risk of stroke (Gupta et al., 2012; Reinhard et al., 2014), none of the subjective assessments based on degree of stenosis and flow were significantly correlated with the risk of stroke; the strongest correlation was shown by CVR. We are in accord with previous authors (Bang et al., 2008; Sheth et al., 2016; Tan et al., 2016) that hold that this CVR correlation occurs because it is related to the presence and extent of functioning collateral blood flow.

### Incomplete Compensation From Collateral Blood Flow

Two additional observations from this study are notable. First, the CVR in patients with >70% stenosis whose CVR was in the “normal” range was almost all in the lower range of normal (Figure 3), an aspect not appreciated until z scoring of the CVR normalized it to a healthy cohort (Sobczyk et al., 2015). This observation follows a general principle in physiology of “incomplete compensation for an active biological disturbance that alters normal equilibrium.” This reduced range of response was discernable because the stimulus was standardized and was compared to the normal range. This finding suggests that no matter how well the cerebrovascular system is collateralized, compensation in patients will never be as good as in healthy subjects.

### Clinical Implications

The second additional observation was that many patients with <50% stenosis also had ipsilateral mean CVR z scores below the normal range (Figure 3), further weakening the widely assumed relationship between the degree of stenosis and adequacy of perfusion reserve. Reductions in CVR in some of these patients who may have no pressure gradient across the stenosis at rest may be attributed to the development of a pressure gradient with the increased flow and turbulence induced by hypercapnia. The normal blood flow delivery at rest would not provide the stimulus for the induction of collateral vessel formation. Thus, the presence or absence of collateral flow, rather than simply the degree of stenosis, may determine the risk of stroke (Gupta et al., 2012; Reinhard et al., 2014), the size and distribution of the infarct (Verma et al., 2015), and the outcome of revascularization (Lima et al., 2010; Smeeing et al., 2016).

#### Implication for Clinical Trials

The presence or absence of a functioning collaterome (Liebeskind, 2015) affects the outcome of CAS patient treatment. In the case of best medical treatment (BMT), the benefits may be due to a well-functioning “collaterome” rather than the medical treatment itself. In the absence of a functional collaterome, BMT would provide added benefit only to the extent that it prevents embolic phenomena and progression of vascular occlusions. In the presence of an effective collaterome, surgical revascularization is a redundant protection, yet the patients incur the other risks associated with anesthesia and surgery. These potential confounding conditions are applied when degree of stenosis alone is the criterion for randomization. We suggest that a better basis for equipoise is to account for the functional state of the collaterals prior to randomization of treatment. Surgical or mechanical revascularization may also not be a suitable intervention without verification of hemodynamic benefit. This too can be indicated and quantified (Sobczyk et al., 2016) as an improvement in CVR.

### CVR and Penumbra

The presence or absence of a functioning collaterome also determines the state of the penumbral volume during and after stroke. An assessment of the collateral flow to the penumbra would inform the choice of therapeutic options, timing of interventions, and prognosis, and thereby likely affect outcome. However, during the acute phase of stroke, CVR is time consuming and risks delaying definitive treatment. Hypercapnia risks vasodilating healthy parts of the brain, which would further reduce the perfusion pressure to the penumbra (“vascular steal”; Fisher et al., 2018). Nevertheless, it may be worth investigating if the benefits of a brief, standardized moderate hypercapnic stimulus equivalent to a single nocturnal hypopnea (to which patients with stroke are particularly prone; Redline et al., 2010) outweigh the risks of CVR for assessment of the collaterome.

### Study Strengths and Limitations

This study was a retrospective, single center, case series design. All patients meeting inclusion criteria were recruited from studies incorporating CVR but were otherwise unselected. We have used a previously generated atlas of normal CVR values as a common reference standard for our studies. This atlas consisted of 46 people who were mostly younger than our cohort. However, we have examined the CVR in the atlas by age and sex (Sobczyk et al., 2015) and found that there were no discernable trends. In any event, the atlas values were applied uniformly and, therefore, function as a reference standard for all patients.

An important aspect of this study was the minimization of CVR variability as a result of implementing a uniform, repeatable hypercapnic normoxic stimulus for all subjects. This implementation enabled the scoring of each voxel CVR in each patient by reference to a normal range. Otherwise, with the range of CVR at each extent of CAS, we would not have been able to objectively discern sub-groups of patients for comparison. Automation of the stimulus administration ensures compatibility of the data between patients in an institution and between institutions (Reinhard et al., 2014; Smeeing et al., 2016) and enables the study to be precisely replicated by others.

The ROC analysis was performed to give a statistical overview of the BOLD MRI-CO_2_ z score diagnosis of impaired CVR using grade of stenosis as a reference standard. However, using grade of stenosis as the “truth data” for the ROC analysis has its limitations and must be interpreted with caution. A strong result may indicate that CVR is unnecessary. However, as Figures 2B,C indicate, there are a number of individuals who have high grade stenosis but preserved CVR. Relying on the degree of stenosis alone would exclude these subsets of individuals. A weak ROC result may indicate that either measure (CVR mean z scores or degree of stenosis) has large measurement error rather than indicate that CVR has more information than degree of stenosis. As Figure 3 presents, CVR does have more information than degree of stenosis alone. Ultimately, future work will require a separate study to test the ability of CVR to better predict stroke than degree of stenosis.

## Conclusion

While CVR was related to the degree of stenosis over the patient cohort, it was highly variable across all degrees of stenosis. As such, CVR does not simply reflect the degree of CAS but, by being a measure of the net increase in flow, it reflects the ability of the vasculature to recruit blood flow in response to an increase in demand. Depending on the degree of obstruction of upstream major arteries, such flow can be, at least, partially attributable to collateral blood flow pathways. As methods of assessment of collaterals vessels improve, future work will need to continue examining the relationship between the structure and function of collateral vessels.

## Data Availability Statement

All datasets presented in this study are included in the article/Supplementary Material.

## Ethics Statement

The studies involving human participants were reviewed and approved by University Health Network. The patients/participants provided their written informed consent to participate in this study.

## Author Contributions

OS, DMM, AC, LV, JD, JF, and DJM all contributed to the design and conceptualization of the study. OS, KS, LM, and JP were involved with data collection. OS, AC, JD, and JF were involved in the data analysis. OS, JF, and DJM drafted the manuscript. All authors participated in the feedback and writing process following the initial drafting of the manuscript. All authors contributed to the article and approved the submitted version.

### Conflict of Interest

JF and DJM contributed to the development of the automated end-tidal targeting device, RespirAct™ (Thornhill Medical Inc.) used in this study and have equity in the company. OS and JD receive salary support from TRI. TRI provided no other support for the study.

The remaining authors declare that the research was conducted in the absence of any commercial or financial relationships that could be construed as a potential conflict of interest.

## Footnotes

^1^http://afni.nimh.nih.gov/afni

^2^http://www.fil.ion.ucl.ac.uk/spm/software/spm8

^3^www.systatsoftware.com
