# Supplementary material for: Cerebrovascular Reactivity Assays Collateral Function in Carotid Stenosis
Source: Front Physiol. 2020 Sep 1;11:1031. doi: 10.3389/fphys.2020.01031 (PMC7528398; doi:10.3389/fphys.2020.01031)
Supplement: Supplementary file 1 [file Data_Sheet_1.docx]

**Supplemental Material**

**Supplemental** **Table 1.** Patient demographics, diagnosis of degree of stenosis and mean CVR and z score hemispheric data. CVR, cerebrovascular reactivity; ICA, internal carotid artery; L, Left; R, Right.

| **Case No.** | **Age** | **Sex** | **Angiographic**  **Findings** | **Side** | **Mean Hemispheric Score** | | | |
| --- | --- | --- | --- | --- | --- | --- | --- | --- |
|  |  |  |  |  | **Gray Matter** | | **White Matter** | |
|  |  |  |  |  | **CVR** | **Z score** | **CVR** | **Z score** |
| 1 | 67 | M | bilateral ICA occlusion | L | 0.0745 | -1.3385 | 0.063 | -1.1362 |
|  |  |  |  | R | 0.0785 | -1.318 | 0.0672 | -1.0742 |
| 2 | 76 | M | bilateral ICA occlusion | L | 0.159 | -0.5977 | 0.1247 | -0.3548 |
|  |  |  |  | R | 0.1312 | -0.8383 | 0.0958 | -0.6388 |
| 3 | 48 | M | RICA near occlusion and >70% LICA stenosis | L | 0.2317 | 0.1179 | 0.2004 | 0.8343 |
|  |  |  |  | R | 0.087 | -1.2858 | 0.0679 | -1.0411 |
| 4 | 40 | F | bilateral ICA occlusion | L | 0.1545 | -0.511 | 0.1169 | -0.4277 |
|  |  |  |  | R | 0.0954 | -1.1977 | 0.0558 | -1.3491 |
| 5 | 74 | M | bilateral >50% ICA stenosis | L | 0.0377 | -1.6397 | 0.0115 | -1.9705 |
|  |  |  |  | R | 0.0861 | -1.2256 | 0.04722 | -1.3372 |
| 6 | 69 | M | RICA 78% stenosis and LICA occlusion | L | 0.0094 | -1.9459 | 0.0043 | -2.0303 |
|  |  |  |  | R | 0.0133 | -1.9491 | 0.009 | -1.8765 |
| 7 | 80 | M | RICA near occlusion and 90% LICA stenosis | L | 0.1991 | -0.2909 | 0.1465 | -0.1195 |
|  |  |  |  | R | 0.1844 | -0.4379 | 0.1154 | -0.5586 |
| 8 | 50 | F | bilateral ICA occlusion | L | 0.0916 | -1.1981 | 0.0502 | -1.371 |
|  |  |  |  | R | 0.084 | -1.2014 | 0.0513 | -1.3133 |
| 9 | 66 | M | RICA occlusion and LICA <50% stenosis | L | 0.173 | -0.4802 | 0.1157 | -0.608 |
|  |  |  |  | R | 0.0235 | -1.9468 | -0.0084 | -2.2742 |
| 10 | 85 | M | RICA 90% stenosis and LICA 70% stenosis | L | 0.1585 | -0.4749 | 0.1548 | 0.2313 |
|  |  |  |  | R | 0.0837 | -1.1712 | 0.0964 | -0.576 |
| 11 | 76 | F | RICA near occlusion and LICA 50-70% stenosis | L | 0.1347 | -0.8492 | 0.1072 | -0.578 |
|  |  |  |  | R | 0.123 | -0.9187 | 0.0961 | -0.636 |
| 12 | 78 | M | bilateral ICA occlusion | L | 0.0896 | -1.2907 | 0.0726 | -1.0541 |
|  |  |  |  | R | 0.0875 | -1.3009 | 0.0708 | -0.9305 |
| 13 | 50 | M | LICA occlusion and R <50% stenosis | L | 0.1722 | -0.6483 | 0.079 | -1.0898 |
|  |  |  |  | R | 0.2799 | 0.4632 | 0.1838 | 0.6977 |
| 14 | 60 | M | LICA occlusion and R <50% stenosis | L | 0.05182 | -1.58552 | 0.02399 | -1.77191 |
|  |  |  |  | R | 0.21111 | 0.001 | 0.177001 | 0.588503 |
| 15 | 59 | M | LICA >70% stenosis and RICA <50% stenosis | L | -0.119773 | -3.0741 | -0.101313 | -3.28307 |
|  |  |  |  | R | 0.0506775 | -1.55025 | 0.0160046 | -1.74263 |
| 16 | 50 | M | LICA occlusion and R <50% stenosis | L | 0.210439 | -0.235379 | 0.117606 | -0.742141 |
|  |  |  |  | R | 0.263366 | 0.371035 | 0.173853 | -0.119773 |
| 17 | 65 | M | LICA >50% stenosis and RICA <50% stenosis | L | 0.0956876 | -1.15856 | 0.0588608 | -1.37321 |
|  |  |  |  | R | 0.114382 | -1.02563 | 0.0708814 | -1.13907 |
| 18 | 52 | F | LICA near occlusion and RICA <50% stenosis | L | 0.0412604 | -1.80328 | -0.0105465 | -2.44979 |
|  |  |  |  | R | 0.221213 | -0.0127601 | 0.131305 | -0.399709 |
| 19 | 36 | F | LICA >50% stenosis and RICA <50% stenosis | L | 0.142004 | -0.85532 | 0.0906352 | -0.91542 |
|  |  |  |  | R | 0.238588 | 0.0873258 | 0.167328 | 0.314235 |
| 20 | 53 | M | LICA occlusion and R <50% stenosis | L | 0.0692738 | -1.53454 | 0.0206669 | -1.80321 |
|  |  |  |  | R | 0.228165 | -0.0912502 | 0.150684 | 0.0907245 |
| 21 | 18 | F | LICA <50% stenosis and RICA <50% stenosis | L | 0.149488 | -0.742953 | 0.102389 | -0.729441 |
|  |  |  |  | R | 0.288952 | 0.789569 | 0.197773 | 0.795367 |
| 22 | 56 | F | LICA >70% stenosis and RICA <50% stenosis | L | 0.227167 | -0.055705 | 0.156768 | 0.0013135 |
|  |  |  |  | R | 0.230835 | -0.0125311 | 0.157245 | 0.163141 |
| 23 | 59 | M | LICA occlusion and R <50% stenosis | L | -0.0515057 | -2.64922 | -0.0849374 | -3.50837 |
|  |  |  |  | R | 0.185984 | -0.373182 | 0.115388 | -0.527785 |
| 24 | 52 | F | LICA occlusion and R <50% stenosis | L | -0.106046 | -3.00333 | -0.101127 | -3.49886 |
|  |  |  |  | R | 0.219115 | -0.076262 | 0.152109 | -0.0013541 |
| 25 | 67 | F | LICA occlusion and R <50% stenosis | L | -0.0140179 | -2.24143 | -0.0289044 | -2.54484 |
|  |  |  |  | R | 0.243109 | 0.22455 | 0.175985 | 0.511842 |
| 26 | 75 | M | LICA occlusion and R <50% stenosis | L | 0.175469 | -0.533956 | 0.13561 | -0.191821 |
|  |  |  |  | R | 0.189202 | -0.305399 | 0.153649 | 0.219428 |
| 27 | 55 | M | LICA occlusion and R <50% stenosis | L | 0.176041 | -0.496199 | 0.122687 | -0.43152 |
|  |  |  |  | R | 0.175636 | -0.515157 | 0.118268 | -0.377812 |
| 28 | 31 | F | LICA occlusion and R <50% stenosis | L | 0.0228425 | -1.91382 | -0.0200515 | -2.60843 |
|  |  |  |  | R | 0.0836839 | -1.08981 | 0.0372469 | -1.67936 |
| 29 | 70 | M | LICA occlusion and R <50% stenosis | L | -0.108507 | -3.00906 | -0.0892163 | -3.39856 |
|  |  |  |  | R | 0.115958 | -0.958628 | 0.0843856 | -0.863445 |
| 30 | 71 | M | RICA occlusion and LICA <50% stenosis | L | 0.123657 | -0.999016 | 0.0731425 | -1.16947 |
|  |  |  |  | R | -0.00181862 | -2.14306 | -0.00723056 | -2.15237 |
| 31 | 71 | M | RICA occlusion and LICA <50% stenosis | L | 0.176943 | -0.46597 | 0.141322 | -0.0744229 |
|  |  |  |  | R | 0.107673 | -1.19186 | 0.0599033 | -1.21551 |
| 32 | 58 | M | RICA occlusion and LICA <50% stenosis | L | 0.187761 | -0.37986 | 0.137634 | -0.13376 |
|  |  |  |  | R | -0.0113113 | -2.14714 | -0.00848441 | -2.13543 |
| 33 | 68 | F | RICA >50% stenosis and LICA <50% stenosis | L | 0.176797 | -0.480733 | 0.115064 | -0.529814 |
|  |  |  |  | R | 0.166886 | -0.583093 | 0.0858216 | -0.921118 |
| 34 | 88 | M | RICA 80% stenosis and LICA 30% stenosis | L | 0.228932 | 0.376485 | 0.191604 | 0.69235 |
|  |  |  |  | R | 0.180225 | -0.0334647 | 0.131734 | -0.0691615 |
| 35 | 34 | M | RICA >70% stenosis and LICA <50% stenosis | L | 0.207623 | -0.207392 | 0.142766 | -0.207505 |
|  |  |  |  | R | 0.120175 | -1.07532 | 0.0573594 | -1.34085 |
| 36 | 48 | F | RICA 98% stenosis and LICA <50% stenosis | L | 0.312267 | 0.918429 | 0.247708 | 1.38064 |
|  |  |  |  | R | 0.0329009 | -1.83081 | -0.00689326 | -2.22071 |
| 37 | 56 | M | RICA and LICA >50% stenosis | L | 0.196799 | -0.241654 | 0.135051 | -0.124339 |
|  |  |  |  | R | -0.0591473 | -2.58579 | -0.0463847 | -2.63078 |
| 38 | 84 | M | RICA occlusion and LICA <50% stenosis | L | 0.0439911 | 0.0224443 | -1.78067 | -1.83666 |
|  |  |  |  | R | 0.0129524 | -0.0249102 | -2.07158 | -2.47627 |
| 39 | 74 | M | RICA near occlusion and LICA <50% stenosis | L | 0.187856 | -0.433435 | 0.14133 | -0.147484 |
|  |  |  |  | R | 0.0759253 | -1.39984 | 0.0528422 | -1.3841 |
| 40 | 32 | F | RICA >50% stenosis and LICA <50% stenosis | L | 0.156577 | -0.760634 | 0.0770192 | -1.2265 |
|  |  |  |  | R | 0.118539 | -1.06318 | 0.0527486 | -1.43415 |
| 41 | 61 | F | RICA occlusion and LICA <50% stenosis | L | 0.163799 | -0.524912 | 0.120516 | -0.451576 |
|  |  |  |  | R | -0.0411023 | -2.49667 | -0.0419166 | -2.70394 |
| 42 | 45 | M | RICA occlusion and LICA <50% stenosis | L | 0.219698 | -0.201476 | 0.154568 | -0.0319368 |
|  |  |  |  | R | 0.173894 | -0.588827 | 0.100948 | -0.730095 |
| 43 | 66 | M | RICA occlusion and LICA <50% stenosis | L | 0.195456 | -0.331653 | 0.136149 | -0.240476 |
|  |  |  |  | R | 0.140508 | -0.855387 | 0.0778102 | -1.03446 |
| 44 | 83 | M | RICA occlusion and LICA <50% stenosis | L | 0.114112 | -1.05805 | 0.0704989 | -1.14464 |
|  |  |  |  | R | -0.0309368 | -2.45567 | -0.0473692 | -2.83555 |
| 45 | 48 | M | RICA occlusion and LICA <50% stenosis | L | 0.186217 | -0.38289 | 0.141811 | -0.094577 |
|  |  |  |  | R | -0.00248662 | -2.1408 | -0.0255959 | -2.47642 |
| 46 | 81 | F | RICA occlusion and LICA <50% stenosis | L | 0.205354 | -0.251988 | 0.153947 | 0.141009 |
|  |  |  |  | R | 0.164627 | -0.546672 | 0.0864867 | -0.900189 |
| 47 | 63 | M | RICA occlusion and LICA <50% stenosis | L | 0.281316 | 0.587412 | 0.228628 | 1.1697 |
|  |  |  |  | R | 0.0853781 | -1.31554 | 0.0236027 | -1.82765 |
| 48 | 53 | M | RICA occlusion and LICA <50% stenosis | L | 0.207636 | -0.0784722 | 0.155241 | 0.117388 |
|  |  |  |  | R | 0.0919921 | -1.21618 | 0.0795889 | -0.800683 |
| 49 | 22 | M | bilateral ICA occlusion | L | 0.249905 | 0.238468 | 0.165347 | 0.0776528 |
|  |  |  |  | R | 0.135583 | -0.910092 | 0.0757833 | -1.075 |
| 50 | 77 | F | RICA 50% stenosis and LICA <50% stenosis | L | 0.257351 | 0.251967 | 0.198377 | 0.719028 |
|  |  |  |  | R | 0.064723 | -1.46342 | 0.0515674 | -1.25527 |
| 51 | 83 | F | RICA occlusion and LICA 50-70% steosis | L | 0.143691 | -0.777715 | 0.100177 | -0.810632 |
|  |  |  |  | R | 0.0693416 | -1.48242 | 0.0439222 | -1.48228 |
| 52 | 48 | F | RICA occlusion and LICA <50% stenosis | L | 0.16545 | -0.683058 | 0.113976 | -0.583852 |
|  |  |  |  | R | 0.142663 | -0.896294 | 0.0793025 | -1.04126 |
| 53 | 77 | M | RICA near occlusion and LICA >70% stenosis | L | 0.188499 | -0.389451 | 0.14649 | -0.0539674 |
|  |  |  |  | R | 0.0673614 | -1.52873 | 0.0411569 | -1.51324 |
| 54 | 64 | F | RICA near occlusion and LICA <50% stenosis | L | 0.233679 | -0.112474 | 0.145592 | -0.232001 |
|  |  |  |  | R | 0.202374 | -0.323249 | 0.120244 | -0.484285 |
| 55 | 53 | M | RICA occlusion and LICA >70% stenosis | L | 0.129333 | -0.949093 | 0.0871953 | -0.895821 |
|  |  |  |  | R | -0.0349223 | -2.40204 | -0.0373376 | -2.63129 |
